# Genetic and epigenetic methylation defects and implication of the *ERMN* gene in autism spectrum disorders

**DOI:** 10.1038/tp.2016.120

**Published:** 2016-07-12

**Authors:** A Homs, M Codina-Solà, B Rodríguez-Santiago, C M Villanueva, D Monk, I Cuscó, L A Pérez-Jurado

**Affiliations:** 1 Departament de Ciències Experimentals i de la Salut, Universitat Pompeu Fabra, Barcelona, Spain; 2 Institut Hospital del Mar d’Investigacions Mèdiques, Barcelona, Spain; 3 Centro de Investigación Biomédica en Red de Enfermedades Raras, Barcelona, Spain; 4 Quantitative Genomic Medicine Laboratories, S.L., Barcelona, Spain; 5 Center for Research in Environmental Epidemiology, Barcelona, Spain; 6 Consorcio de Investigación Biomédica de Epidemiología y Salud Pública, Barcelona, Spain; 7 Cancer Epigenetics Group, Institut d'Investigació Biomedica de Bellvitge, Hospital Duran i Reynals, Barcelona, Spain

## Abstract

Autism spectrum disorders (ASD) are highly heritable and genetically complex conditions. Although highly penetrant mutations in multiple genes have been identified, they account for the etiology of <1/3 of cases. There is also strong evidence for environmental contribution to ASD, which can be mediated by still poorly explored epigenetic modifications. We searched for methylation changes on blood DNA of 53 male ASD patients and 757 healthy controls using a methylomic array (450K Illumina), correlated the variants with transcriptional alterations in blood RNAseq data, and performed a case–control association study of the relevant findings in a larger cohort (394 cases and 500 controls). We found 700 differentially methylated CpGs, most of them hypomethylated in the ASD group (83.9%), with *cis*-acting expression changes at 7.6% of locations. Relevant findings included: (1) hypomethylation caused by rare genetic variants (meSNVs) at six loci (*ERMN*, *USP24*, *METTL21C*, *PDE10A*, *STX16* and *DBT*) significantly associated with ASD (*q*-value <0.05); and (2) clustered epimutations associated to transcriptional changes in single-ASD patients (*n*=4). All meSNVs and clustered epimutations were inherited from unaffected parents. Resequencing of the top candidate genes also revealed a significant load of deleterious mutations affecting *ERMN* in ASD compared with controls. Our data indicate that inherited methylation alterations detectable in blood DNA, due to either genetic or epigenetic defects, can affect gene expression and contribute to ASD susceptibility most likely in an additive manner, and implicate *ERMN* as a novel ASD gene.

## Introduction

Autism spectrum disorders (ASD; OMIM 209850) are a group of early-onset neurodevelopmental disorders diagnosed on the basis of two symptom areas: social communication impairments, and restricted repetitive behaviors, interests or activities (DSM-V). The currently estimated prevalence of ASD is 1/42 males and 1/189 females aged 8 years, after a documented prevalence increase during the past two decades.^1^ There is strong evidence for a genetic etiology of ASD with heritability ranging from 50 to 90%.^2, 3^ However, despite remarkable advances driven by genetics and genomics leading to the identification of single-gene mutations, copy-number changes and double hit models, the etiology of around 70% of ASD cases still remains unknown.^4, 5^ This relatively poor detection rate could be explained in part by the high phenotypic and genetic heterogeneity of ASD with multiple inheritance models, including monogenic, oligogenic and polygenic with gene–environment interactions.^6, 7, 8^ In addition, most genomic studies to date have focused on the analysis of sequence and copy-number changes in the coding part of the genome, therefore, missing putative epimutations and other changes located elsewhere in the genome.

Epigenetics comprises DNA methylation, long non-coding RNA and histone post-translational modifications, which act as regulatory mechanisms of gene expression. Epigenetic aberrations have been associated with a wide range of diseases including ASD.^9, 10^ Several single-gene disorders commonly associated with autism (that is, Fragile-X^11^ and Rett syndromes^12^ show multiple epigenetic deregulations, and recurrent genomic alterations in chromosomal regions subjected to imprinting (chr7q, 15q (ref. 13, 14)) commonly manifest as ASD. Other epigenetic defects secondary to mutations in genes such as *UBE3A*^15^ and *OXCTR*,^16^ as well as altered CpG methylation in genes such as *RORA, BCL1* ref. 17) and *EN-2*,^18^ have also been implicated in autism. Therefore, methylation and imprinting alterations associated or not to genetic anomalies, may be responsible for additional cases of ASD. Although epigenetics is still a poorly explored field in ASD, the current genome-wide technologies permit to assess DNA methylome in the available tissues. To date, several small-size case–control studies have addressed methylomic abnormalities in ASD searching for differences in twin pairs discordant for the disease on cell line or blood DNA,^17, 19^ investigating DNA from different brain regions,^20, 21^ or looking for effects of advanced maternal age on buccal epithelium as a surrogate of ectodermal cells.^22^ Methylation analyses of promoter regions of genes in blood DNA failed to detect relevant changes in an initial study (analyzing 1505 regions in 70 patients),^23^ while reporting hypermethylation at *ENO2* in 14.5% ASD cases (analyzing 16,000 regions in 131 patients).^24^

In this study we aimed to identify methylation aberrations in blood DNA of a cohort of ASD patients in a search for testable epigenetic biomarkers contributing to ASD etiology. Functional consequences were analyzed by RNAseq of blood RNA, integrating the analysis of epigenetic variants and their transcriptional consequences. Finally, we performed a case–control study of the resulting rare variants using an independent and larger ASD sample in order to increase the power and significance of the associations, as well as a screening for mutations affecting the coding sequence in the previously identified genes.

## Materials and methods

### Study population

We selected 53 unrelated idiopathic ASD male patients, aged from 2 to 14 years (Supplementary Table 1) and referred from several hospitals of the Spanish Health System. All individuals had a diagnosis of ASD based on clinical and psychological evaluations (DSM-IV and ADI-R test). Genetic and biochemical testing for comorbid conditions (Fragile-X and some metabolic disorders), and molecular karyotyping (aCGH or SNP array) were negative in all cases. The study was approved by the ethical committee of the centers involved (CEIC-Parc Salut Mar), and written informed consent was obtained from the parents or legal tutors.

Healthy controls consisted of 10 age-matched Spanish male children analyzed simultaneously. Extraction of DNA and RNA was performed from peripheral blood with the Blood DNA extraction kit (Qiagen, Hilden, Germany), and RNA was isolated from lymphocytes using a ficoll density-gradient (STEMCELL Technologies, Vancouver, BC, Canada), according to the manufacturer’s instructions.

Additional methylation data from 91 controls (males of a wide age range) were obtained from the Center for Research in Environmental Epidemiology (CREAL) and the Bellvitge Institute for Biomedical Research (IDIBELL),^25, 26^ as well as from 656 controls (males and females of European ancestry aged from 19 to 101 years) available through GEO (GSE40279). All 757 control samples were obtained from whole blood, except for 5 from cord blood.

In addition, DNA methylation data (blood, 415 ASD and 405 healthy siblings, males, 6 to 25 years old) obtained with the HumanMethylation27 platform (Illumina, San Diego, CA, USA) were downloaded from dbGAP database (accession, phs000619) and probes shared by both platforms were compared.

### Methylomic analyses

The DNA was bisulfite converted and hybridized onto Illumina HumanMethylation450K BeadChips (Illumina) at the Spanish National Genotyping Centre (Barcelona, Spain), according to the manufacturer’s protocol. Samples were placed randomly through the chips and a technical replicate was included in each chip. Raw data were obtained with Genome Studio (Illumina; v1.8) and quality controls included technical replicates correlation, detection *P*-value filtering and a bisulfite conversion control. The obtained correlation Pearson’s mean coefficient values were over 0.903 and the Spearman’s coefficient (rho) over 0.962. Detection *P*-value was filtered removing probes with *P*-values over 0.01 and sample bisulfite conversion passed the intensity threshold of 4000 as indicated in manufacturer’s instructions.

We obtained the beta-values for each CpG, ranging from 0 (unmethylated) to 1 (completely methylated). Simple scaling normalization was applied using the HumMeth27QCReport^27^ and LUMI^28^ packages in R.

Differential methylation (DM) analyses were performed using Limma R Package,^29^ applying eBayes and linear BH-FDR adjustment; *q*-value was set at 0.01. Probes with low-quality detection signal in >5% of the samples, with cross-reactive hybridization,^30^ or harboring known single-nucleotide polymorphisms (SNPs; minor allele frequency (MAF)>0.005) within the CpG±5nt were removed (dbSNPv137.2 and CEU 1000-Genomes databases). We used two different approaches to perform DM analyses: we first compared the ASD group versus the control group, and then, based on the assumption that genetic alterations in ASD are highly heterogeneous, we compared each patient individually versus the control group. Following the individual approach, we curated the results discarding differentially methylated CpGs (DMCpGs) altered in any of the control samples, and those with values below the absolute methylation difference threshold (Delta Beta value⩾|1.8|, representing 18% of difference). We prioritized probes showing methylation-status changes as previously reported,^31^ being the hypomethylated status with beta-values <0.3, hypermethylated with beta values >0.7 and intermediate beta in the 0.3–0.7 range. After the first DM analysis, we performed a second DM analysis including new healthy control data from an epidemiology study (*n*=91, Spanish, males, wide age range), applying the same statistical analysis and filtering criteria, except for a less-strict fold change (under 1.5 or 15%). Finally, we compared the resulting DMCpGs with a publicly available control data set (GSE40279; *n*=656 Hispanic and CEU, males and females, wide age range) and discarded probes altered in this control set with a frequency >0.005, considered as relatively common epigenetic variants. We obtained DMCpGs and differentially methylated regions (DMRs). We defined DMRs as regions with more than one significant DMCpGs close to each other (using our strict filter of significant difference >18% at each DMCpG). We also used the COMB-P^32^ algorithm to detect more subtle methylation changes in selected windows of 1000 kb and weighted for 50-bp windows with a nominal *P*-value <0.01. Manhattan clustering and principal component analyses were performed using R.

We also performed subset-quantile within array normalization (SWAN) to correct type1/type2 errors using minfi tool,^33^ considering an absolute methylation difference of 7% as previous studies.^20, 22^ Then, the same pipeline as above was applied to define the final DMCpGs.

### DMCpGs validation

We performed genetic and epigenetic validations of selected altered DMCpGs. Parental samples of ASD probands were also analyzed. The same DNA samples were subjected to PCR amplification and Sanger sequencing to analyze the sequence, and to pyrosequencing and BS-PCR direct sequencing^34^ to analyze the methylation levels quantitatively or qualitatively. Pyrosequencing analyses were performed using PyroMark Q96 (Qiagen, Hilden, Germany) following the manufacturer’s instructions and the results were analyzed with the software provided. Primer sequences for pyrosequencing are available upon request. Pyrosequencing was also used to replicate the analysis in additional samples. Methylation was also assessed qualitatively by BS-PCR direct sequencing.^34^ Primers were designed with Methyl Primer Express v.1. Software (Applied Biosystems, Carlsbad, CA, USA) and PCR conditions are available on request.

### Analysis of *cis*-acting elements on DMRs

We searched for putative genetic variants in the flanking intervals of the DMRs. We designed primers at both sides of the DMRs (including ~1 kb in each side), and performed Sanger sequencing of the PCR amplified products on specific samples. We also used multiple-ligation probe-amplification (MLPA) with probes located next to the DMCpGs on these regions using standard protocols and analytical methods. Primers and probes were designed as explained above and are available upon request.

### Large-scale genotyping

In order to define a putative association of the identified rare variants in ASD patients, we genotyped 32 rare SNVs (Supplementary Table 2) in an additional set of 394 ASD samples (Spanish and ECACC collection) and 500 Spanish controls by Sequenom (San Diego, CA, USA). We used 250 ng of DNA to perform the Sequenom assay, which was conducted at Universitat de Valencia (Valencia, Spain). To compute the statistical association we included all the data (450K, 27k and Sequenom) and compared allelic frequencies. Fisher’s exact test and correction by false discovery rate (FDR) were performed using R (fisher.test function and *q*-value^35^).

### RNA sequencing

To identify the functional consequences of methylation alterations we performed an RNAseq study in 29 out of the 53 studied samples, from which we obtained good quality RNA from blood (RIN>7). The RNA library was prepared and sequenced (paired-end 100-bp reads) using Illumina HiSeq 2000 (Illumina) and sequence reads aligned (build hg37) using TopHat^36^ and Bowtie.^37^ Expression was quantified by htseqcount in Conditional Quantile Normalization counts.^38^ Data were corrected for batch effect by Combat algorithm (SVA package R).^39^ We assessed case–control differential expression of the genes flanking 50 kb upstream and downstream the DMCpG, distance used in similar studies.^40, 41, 42^ We selected altered genes by *Z*-score, selecting *Z*-scores over 2 (*P*-value <0.05) or 1.5 if two genes were altered in the same region, as recommended in expression analysis.^43^

### Targeted NGS sequencing

The coding region of five selected genes (*ERMN, STX16, SMG7-AS1, PIK3CD and ZCCHC9*) was included in a targeted resequencing panel analyzing 279 additional idiopathic ASD cases and 105 controls. Samples were fragmented and processed with the TruSeq DNA Sample Preparation kit (Illumina). The libraries were captured by the NimbleGen SeqCap EZ custom Library (Hofman-La Roche, Basel, Switzerland). Quality control was assessed using FastQC tool,^44^ adapter sequences were trimmed using Trim Galore^45^ and mapping was performed using BWA.^46^ GATK^47^ was used for variant calling and Annovar^48^ for functional annotation. Rare mutations predicted deleterious (loss of function or missense predicted damaging with the CONDEL program^49^) were selected. We also analyzed exome data of ASD patients available from dbGAP (accession=phs000482.v1.p1 and phs000298.v1.p1*; n*=931) in order to define the number of deleterious mutations in selected genes. To define whether the burden of deleterious mutations is higher in ASD, we use exome data from controls in the EXAC database,^50^ and compared the mutational load in cases and controls (Fisher’s test FDR corrected).

### Gene ontology analysis

We searched for enriched pathways in DAVIDGo database,^51^ and for over-represented functions using CPDB^52^ for all the genes associated to the DMCpGs, as well as for the subset of genes with significant association and located in the DMRs.

## Results

### Methylation alterations in blood DNA of ASD

Clustering and principal component analyses showed a random distribution of the methylation data from patients and controls with no common patterns or systemic differences between groups independently of the normalization method applied (Supplementary Figures 1 and 2). A similar result was obtained in the differential methylation analysis comparing cases with controls as groups, with no significant differentially methylated CpG (DMCpGs) identified (*q*-value <0.01).

**Figure 1:**
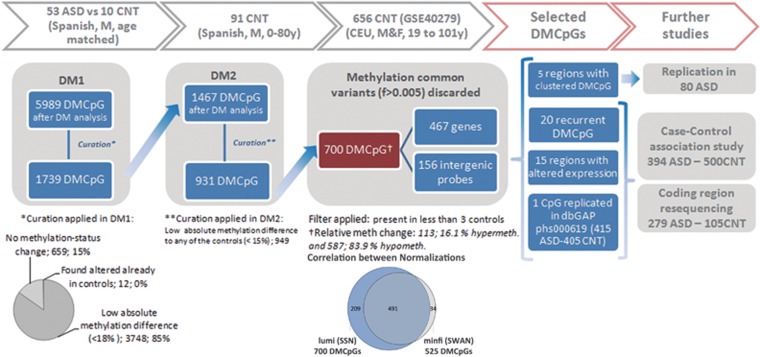
Pipeline followed for the differential methylation analyses with detailed filtering criteria. For the first two comparisons (DM1 and DM2), we established a *P*-value threshold <0.01, whereas in the third comparison (DM3) we searched for rare methylation alterations in a large control cohort (frequency <0.005). We obtained 700 DMCpGs, 156 intergenic probes and 544 of them encompassing 467 genes and 10 miRNA. We selected for further analyses the DMCpGs found in more than one case, including the dbGAP study (accession=phs000619), the differentially methylated regions, and the DMCpGs associated with significant *cis*-acting expression changes. Further studies with patients and controls were performed, for which some public accession data were added to the cohorts (data not added). Venn-diagram: correlation data between two normalization methods showing high concordance. ASD, autism spectrum disorder; C, controls; DM, differential methylation analysis; DMCpGs, differentially methylated CpGs; F, females; M, males.

In order to detect rare methylation defects, we compared each individual ASD patient with the age- and gender-matched control group (*n*=10). After assessing differential methylation with the additional control sets with stringent filtering, we identified 700 DMCpGs present in ASD samples (4 to 33 per sample; Figure 1, and Supplementary Table 3). We observed a high concordance between the two normalization methods (93% of the DMCpGs identified). Only 34 additional DMCpGs were found with minfi (Supplementary Table 4). Therefore, the results were not affected by the normalization approaches. To define the consistency of the identified DMCpGs, we performed sequencing validation on the same DNA samples at 98 sites, including 85 isolated CpGs and 13 plausible DMRs. The validation rate, conceived as true genetic or epigenetic variants, was 85% (84/98), 78 sites corresponding to genetic alterations (meSNVs) and 5 to epigenetic modifications in the DMRs.

Most of the 700 DMCpGs (587; 83.9%) in ASD cases showed a relative hypomethylation compared with controls, and mostly changed from a hypermethylated state to an intermediate state (Beta-values from 30 to 60% Supplementary Figure 3). Regarding genomic location, 544 DMCpGs located within 467 genes and 10 miRNA, whereas 156 DMCpGs were intergenic probes (>1.5 kb from the gene Transcription Starting Sites; Figure 1). We searched for convergence between the detected genes and core ASD-associated genes compiled in SFARI and AutismKb databases (*n*=486 genes), and found 32 genes (6.5% Supplementary Table 5).

Although most DMCpGs were case specific, we found 20 present in two or more ASD patients. Rare SNPs at the CG position (meSNPs) had been described at 9 (45%) of these sites (dbSNP137 and 1000G MAF<0.005), so we assumed the SNPs were the cause of the DMCpG. Validation and segregation analysis of the remaining DMCpGs (*n*=11) revealed that all were also rare single-nucleotide variants (meSNVs) inherited from healthy progenitors (Supplementary Table 2).

We also found five DMRs in four different patients (one patient had two DMRs). Methylation levels were validated, and we discarded underlying genetic changes by dosage analysis with MLPA probes in the region and by sequencing of the flanking intervals. These clustered epimutations were inherited in three cases, two from the father and one from the mother, with identical degree and extension in the parental samples (Table 1 and Supplementary Figures 4–7). A search for DMRs with less-stringent methylation differences using the COMB-P algorithm revealed six additional regions, but they were also found altered in several controls and then not considered as ASD specific.

### Transcriptional consequences of DMCpGs

**Figure 2:**
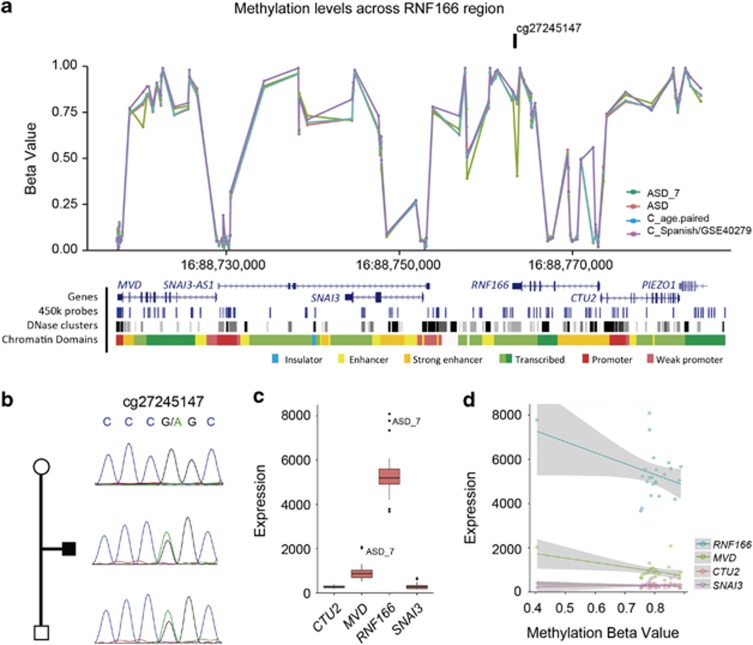
Heterozygous meSNV associated with overexpression of several regional genes at 16q24.3. (**a**) Beta values across the region for the patient (green), the rest of the patients (red), the age-paired (blue) and the Spanish and GEO (GSE:40279; purple) controls sets. Diagram under the graphs shows the genes in the region, 450k array probes, the DHSs and chromatin domains in the region. (**b**) Sanger sequence for the DMCpG showed a paternally inherited meSNV (G>A). (**c**) Expression levels showed overexpression of *RNF166* and *MVD,* and a tendency in *CTU2* (**d**) correlation for methylation values and expression for the genes for all ASD samples. ASD, autism spectrum disorder; C, controls; DHSs, DNAseI hypersensitive sites.

Out of the 476 DMCpGs altered in any of the 29 samples with blood transcriptomic data by RNAseq, we studied expression changes of nearby genes that were expressed in blood (*n*=630). We observed a strong correlation (*Z*-score>2) with expression changes of flanking genes in 7.6% of the loci harboring DMCpGs. Validation and segregation analysis of the 15 DMCpGs outside DMRs revealed that all contained rare inherited meSNVs (Supplementary Table 2, Supplementary Figure 8). Interestingly, a methylation alteration in the promoter of *RNF166* led to a transcriptional alteration of three flanking genes (Figure 2).

**Figure 3:**
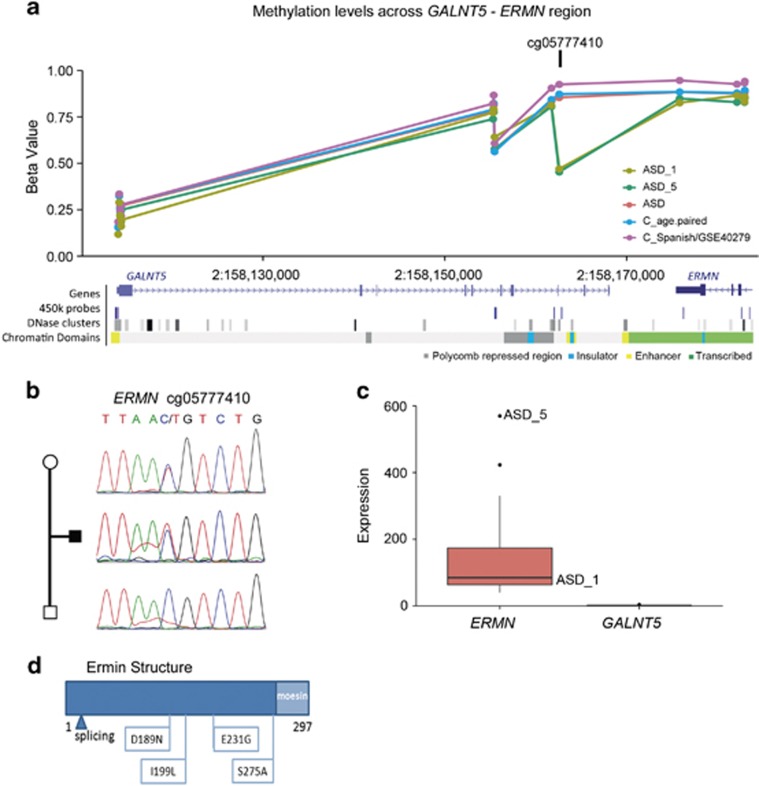
Example of a DMCpGs present in more than one case with associated effects on gene expression. (**a**) Hypomethylation in cg05777410 correlated with *ERMN* overexpression. Beta values for the region showing two individuals (ASD_1 and ASD_5) with DM at a single site in the region, cg05777410; the rest of ASD patients (red), the age-paired (blue) and the Spanish and GEO (GSE:40279; purple) control sets showed a similar pattern. The diagram under the graph shows the genomic context with the *GALNT5* and *ERMN* genes, 450k array probes, DNSs and chromatin domains. (**b**) Sanger sequence of the region in the ASD_5 trio showing a maternally inherited meSNV (C>T in chr2:158162587) at cg05777410. (**c**) Quantitative RNAseq data for *ERMN* and *GALNT5* transcripts showed a significant overexpression of the *ERMN* gene in ASD_5, and ASD_33 compared with the rest of individuals. (**d**) Location of the deleterious mutations at the coding region of Ermin, showing exclusively those detected in ASD patients. ASD, autism spectrum disorder; C, controls.

**Figure 4:**
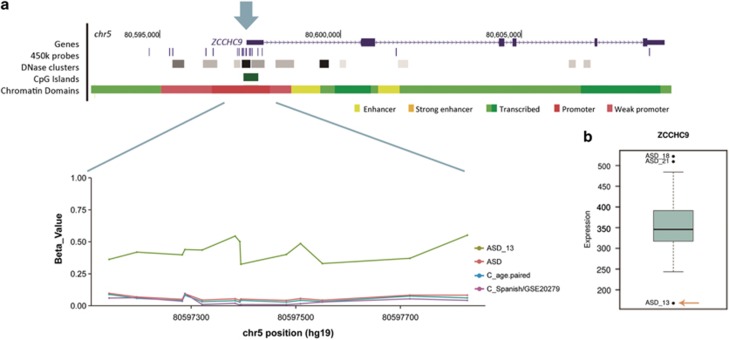
Diagram showing the region and expression for *ZCCHC9* detected DMR. (**a**) Diagram for the region with 13 CpGs altered located in *ZCCHC9* promoter (pointed with an arrow). The diagram shows the gene location, the 450k array probes, the DNAseI hypersensitive clusters, and chromatin domain regions. ASD_13 shows increased methylation levels. (**b**) Expression for all ASD patients for the region. ASD_13 shows a downregulation for the gene. ASD, autism spectrum disorder; C, controls; DMRs, differentially methylated regions.

Regarding the rare meSNVs present in more than one patient, a significant overexpression of the flanking gene(s) was observed in four cases and a downregulation in one. The meSNV causing hypomethylation within *GALNT5* correlated with overexpression of the nearby *ERMN* gene (Table 1 and Figure 3) in one of the cases (ASD_5). Finally, RNAseq also revealed significant changes of the expression levels of flanking genes in 4/5 of the DMRs (Figure 4).

### Rare meSNVs associated to ASD

We first used methylomic (Illumina 27k, Illumina) data from blood DNA of 415 ASD cases and 405 controls as a replication cohort. Only 17 (2.4%) of the 700 DMCpGs were covered by this array. Relative hypomethylation at cg25781162 (*ABCG5*), caused by an inherited meSNP in one of our probands, was also detected in another ASD patient, and absent in controls (Table 1).

To determine whether a selected set of rare meSNVs (found in more than one ASD patient and/or associated with transcriptional effects) were statistically associated to ASD, we genotyped them by Sequenom in an additional 394 ASD and 500 Spanish controls. None of the variants were common in the Spanish control population (MAF<0.002), discarding population-specific variants. Significant association following multiple testing corrections (*q*-value<0.05) was found with five meSNVs (at the loci: *GALNT5-ERMN, USP24, METTL21C*, *PDE10A and DBT*; Table 1). Considering all the data for case–control analyses, six additional meSNVs identified in a single or two ASD patient(s) were absent in all the studied control samples (*n*=935–1713; Table 1).

We also used pyrosequencing in another cohort of ASD patients (*n*=75) to check whether three of the clustered DMRs were present in more cases, but all resulted negative. Therefore, most of these DMRs were patient specific and absent in controls (*n*=757), except for one present in 3 of the 757 controls. Taken together, these patient-specific meSNVs and DMRs are still good candidates that cannot be excluded from being associated with ASD risk, although replication in larger data sets would be required.

### Deleterious mutations in *ERMN* associated to ASD

To identify other putative genetic mutations in some genes whose methylation and expression changes showed association with ASD disease, we performed targeted resequencing of the coding regions of 5 selected genes (*ERMN, STX16, SMG7-AS1, PIK3CD and ZCCHC9*). We completed the screening in additional 279 ASD patients and 105 Spanish controls, used to exclude population-specific variants. No loss-of-function mutations were identified in any of the genes in the ASD cohort, but deleterious missense rare variants were detected. Mutations in *ERMN* were identified in three unrelated ASD patients, one in two cases (c.830G>A/R277Q) and the second in one case (c.823 T>G/S275A) (Supplementary Table 6). We also reanalyzed 931 exomes of ASD patients (previously reported in O’Roak *et al.*^53^ and De Rubeis *et al.*^54^) available from dbGAP and the EXAC repository data to define the mutation load. We identified five additional ASD cases harboring the same R277Q mutation and four additional deleterious mutations in ASD patients (Figure 3 and Supplementary Table 6). In the EXAC repository, a total of 327 predicted pathogenic mutations could be found. By comparison, a significant increase in the mutation load of *ERMN* in ASD with respect to controls was observed (*P*-value=0.046), which is much more significant if we exclude three population-specific variants (*P*-value=7.97E−05). The finding of a higher mutation burden in ASD patients, along with a positive association with a single meSNV at this locus and the overexpression in carriers of the meSNV, strongly support that *ERMN* is implicated in ASD. No significant mutation burden was identified in any of the other screened genes in ASD patients.

### General gene ontology analysis

Gene onthology analysis for the total amount of genes (*n*=467) revealed six significantly (*P*-value<0.05) enriched KEGG pathways (DavidGO; Supplementary Table 7): axon guidance (*n*=10 genes), PIP metabolism (*n*=5 genes), chemokine signaling (*n*=10 genes), focal adhesion (*n*=12 genes), adherence (*n*=6 genes) and tight junctions (*n*=8 genes). Regarding the biological function enrichment annotation chart (CPDB database), we found enrichment in functions such as nervous system development (*n*=73 genes), neuron development (*n*=30), synaptic transmission (*n*=28), cell death (*n*=59 genes) and immune response (*n*=18 genes). In addition, when analyzing all the significant associated regions and the DMRs we found only AMPK signaling pathway significantly enriched (*CAMKK1* and *PIK3CD*).

## Discussion

With the main goal of identifying methylation aberrations as testable epigenetic biomarkers for ASD, we have performed an epigenome-wide association study in blood DNA in a selected cohort of idiopathic ASD patients, and explored the functional consequences on blood transcriptome of a subset. Although ASD is mainly a disorder of neurodevelopment and there are genomic regulatory regions with brain-specific epigenetic states, there is evidence that blood cells can be used as proxies for epigenetic state at the many regions whose epigenetic state is common to multiple tissues.^55^ We used the 450k methylomic array containing selected probes for ~1.7% of the estimated 28 million CpG sites of the human genome. No systemic methylation alterations were identified in this ASD population, as previously shown in other studies of ASD using brain and blood DNA samples.^19, 56^ Despite the small size of our and the previously reported cohorts, the combined data discarded common DMRs in the regions analyzed shared by all ASD patients. In a similar manner to most genetic variants currently associated to ASD, and considering the low-recurrent nature of epigenetic events, low prevalent or individual-specific epimutations would not be detected with this type of group analyses.^56^ We then searched for individual-specific ASD-related variants using a large number of controls (*n*=757), both age matched and adults. We considered that including many controls with a wide age range would provide more power to select ASD-related and significant changes without biased comparisons. Although blood DNA methylation has been shown to vary with aging, the changes are of small magnitude (10% reduction on average) and affecting a small amount of the 450k array probes (2.1–15%).^26, 57^

A total of 700 DMCpGs in 467 genes were detected specifically in the ASD cohort, 13 per patient on average. Interestingly, 83.85% of these DMCpGs showed relative hypomethylation, in agreement with previous reports showing global hypomethylation in blood of ASD patients,^58^ although all clustered regions showed hypermethylation in ASD cases. The integrative study with blood transcriptome allowed the identification of *cis*-acting gene expression effects for 7.6% of the DMCpGs, more commonly overexpression associated to hypomethylation. In support of their potential pathogenic implications, 4.5% of the DMCpGs were also found in ASD patients’ brain,^21^ whereas 6.5% of the genes were ASD-related genes reported by mutation, copy number variant and/or association analyses. Most of the isolated DMCpGs identified were due to meSNVs, and were inherited in all tested cases. The high proportion of meSNVs among DMCpGs was due, at least in part, to the stringent filters applied to focus on the most severe methylation changes (50% or more), expected with mutations at CpG.^30^ Anyhow, heterozygous SNPs at CpGs are clearly implicated in the allele-specific methylation events, being an important class of *cis*-regulatory variants connecting genetics to epigenetics.^59^ The expression changes related to meSNVs can be due to the interference with *trans*-acting regulatory elements of either the methylation alteration itself, the underlying sequence variant or both factors together.

We identified and validated five DMRs in four ASD patients, with *cis*-acting expression effects on neighboring genes documented in four regions. Interestingly, all DMRs were inherited events also found in one of the parents. This germline transmission of epimutations could be explained by reprogramming evasion mechanisms^60^ or by undetected *cis*-acting genetic or genomic mutations affecting methylation of the region.^61^ The hypermethylation of the *SMG7-*antisense and *SMG7* promoter region was paradoxically associated with *SMG7* overexpression in a single patient (Supplementary Figure 6). *SMG7* is involved in nonsense-mediated mRNA decay regulation, a pathway that has also been previously implicated in ASD and related syndromes.^62^ Other DMRs in single-ASD patients affected the regulation of genes involved in the development and function of the nervous system (*PHACTR1*,^63^ *PIK3CD*^64^ and *PKD1*^65^). Methylation deregulation of *PIK3CD* has been shown in ASD brain,^21^ and the gene was associated to schizophrenia.^66^ Similarly, *PKD1* expression was found downregulated in ASD^67^ and also associated to neuropsychiatric diseases, including ASD.^68^

Through an extended case/control association study, we identified six rare meSNVs significantly associated with ASD. The most significant association was detected with a CpG located close to an enhancer and DNAse hypersensitive site between the *GALNT5* and *ERMN* genes (chr2q24.1). Although *GALNT5* is not expressed in blood, *ERMN* was found overexpressed in one patient presenting the rare meSNV. Moreover, by targeted resequencing, we found that *ERMN* showed a higher load of rare damaging mutations in ASD patients. *ERMN* encodes an oligodendroglia-specific cytoskeletal protein (Ermin) involved in myelination, upregulated during active myelination periods in rat axons.^69^ The moesin/actin-binding domain has been shown to be essential for the ability of Ermin to promote arborization and morphological changes in cultured COS-7 cells.^70^ Correct myelination is crucial in ASD and mood disorders.^71, 72, 73^ Interestingly, *ERMN* is located within the boundaries of the *AUTS5* locus, which has repeatedly shown linkage to autism,^74^ language impairment^75^ and IQ.^76^ Moreover, deletions including *ERMN* have been reported in patients with developmental delay and impaired communication,^77^ and *ERMN* expression is lower in epileptic patients brains suggesting also a role in the epileptogenic process.^70^ In fact, gain of function through overexpression and loss of function through deleterious mutations would suggest delicate gene dosage equilibrium.

The other five rare meSNVs significantly associated are also strong ASD candidates affecting relevant genes. (1) *USP24*, encodes a deubiquitinating enzyme that has been associated to Parkinson’s physiopathology.^78^ (2) *PDE10A*, a paternally imprinted gene^79^ highly expressed in specific brain regions, encodes a cyclic nucleotide phosphodiesterase that regulates signal transduction; the gene was found disrupted by a chromosomal translocation in a patient with neurodevelopmental disorder and was proposed as a therapeutic target for cognitive impairment in schizophrenia.^80^ (3) *METTL21C*, encoding a lysine methyltransferase for non-histone proteins, regulates molecular chaperones related to neuroinflamation, such as the Hsp70 found altered in the plasma of some ASD patients.^81^ (4) The meSNV on *TRM13* associated with overexpression of the nearby *DBT*, coding for the transacylase E2 subunit of the branched-chain alpha-keto-acid dehydrogenase complex mutated in patients with autosomal recessive urine disease^82^ and ASD with epilepsy phenotype.^83^ (5) A meSNV showing nominal association was associated with overexpression of *STX16*, which is involved in synaptic vesicle transport, a pathway known to be impaired in ASD.^84^

The remaining rare DMCpGs/meSNVs found in single or few ASD patients need additional studies in larger data sets to confirm a potential association with the phenotype. Some of them have been related to ASD by point mutations (*C15orf62/DNAJC17* and *ADAP1*), linkage studies (*TLE3*), aberrant brain expression (*PRCD, LCP1, RXRB* and *SETD1A*)^67^ and copy number variants (*n*=11 genes). Other genes were involved in relevant functions as neuron differentiation (*SERPINF1*, *TCF12* and *KAL1*), neuron signaling (*C1QL3*) and ubiquitinization *(DNAJC17* and *RAB40B*). Pathways enrichment analysis showed significant results in mechanisms previously related to ASD including axonogenesis,^71^ cell adhesion,^85^ immune chemokine signaling^86^ and PIP signaling.^87^

In summary, this epigenome-wide association study integrated with transcriptome in blood samples revealed significant association of ASD with hypomethylation caused by rare meSNVs at several loci, as well as a few clustered epimutations in single-ASD patients. Thus, methylation alterations detectable in blood DNA, due to either genetic or epigenetic defects, can contribute to ASD susceptibility. Given that most probands harbor more than one methylation variant and all of them are inherited from unaffected parents, their contribution to the phenotype is more likely to be additive, in an oligogenic manner. The study also reveals that *ERMN* is a strong ASD susceptibility gene that can be altered by both rare meSNV and mutations. Additional studies of genome-wide DNA methylation in larger ASD cohorts, integrated with genomic, transcriptomic, proteomic as well as the phenotypic data, are needed to further unravel the complex etiopathogeny of this multifactorial disorder.

## Figures and Tables

**Table 1:** Summary of the relevant DMCpGs found in this study

*REF_ID*	*Position (hg19)*	*Gene*	*No.* *of cases*	*Variant type and segregation (case1/case2 and so on)*	*Meth. avg. ASD/CNT*	*RNAseq expression*	*Association study*			*Reported in ASD*
							*No. of ASD-CNT*	*MAF ASD-CNT*	P*-value (OR (CI))*	
*DMCpGs with statistically significant association*										
cg05964000	chr1:55564130	*USP24*	2	meSNP MAF<0.002, n.a.	0.597/0.891	Normal levels	447–1,349	0.0067–0.0019	0.033^a^; 3.64 (0.92–15.11)	C
cg17937591	chr1:100602503	*DBT*	1	meSNP MAF<0.005; M	0.521/0.891	ASD_23: (OE)	446–1349	0.0045–0.0007	0.037^a^; 6.07 (0.87–67.28)	
cg05777410	chr2:158162587	*GALNT5-ERMN*	2	meSNV; M/M	0.462/0.911	ASD_5: *ERMN* (OE)	445–1348	0.0056–0.0004	0.004^a^; 15.21 (1.7–717.62)	C, R
cg19001113	chr6:166071572	*PDE10A*	3	meSNV; P/M/M	0.53/0.875	Not expressed	443–1349	0.0045–0.0007	0.036^a^; 6.11 (0.87–67.74)	C
cg05491608	chr13:103346962	*METTL21C*	2	meSNP MAF<0.001, n.a.	0.434/0.871	Not expressed	444–1348	0.0079–0.0022	0.023^a^; 3.56 (1.02–12.86)	
cg18904477	chr20:57222576	intergenic	2	meSNP MAF<0.003, n.a.	0.506/0.887	ASD_3: *STX16* (OE)	444–1349	0.0034–0.0004	0.049^b^; 9.13 (0.73–478.77)	
										
*DMCpGs without detected controls in the association study*										
cg03983498	chr1:55107291	*ACOT11*	ASD_4	meSNV; P inh	0.502/0.877	Reduced/OE	446–1349	0.0011–0	0.248; Inf (0.08-Inf)	C
cg24311693	chr6:33147578	*RXRB*	ASD_23	meSNP MAF<0.001; M	0.448/0.805	Reduced/OE	445–1349	0.0011–0	0.248; Inf (0.08-Inf)	C, E
cg04552480	chr16:12209538	*RPS23P6*^c^	ASD_3	meSNP MAF<0.002; P	0.530/0.882	Reduced/OE	445–1349	0.0011–0	0.248; Inf (0.08-Inf)	
cg27245147	chr16:88763611	*MVD*	ASD_7	meSNV; P	0.404/0.829	Reduced/OE	318–954	0.0016–0	0.25; Inf (0.08-Inf)	C
		*RNF166*^c^								mDS
cg20758953	chr22:20762620	*SCARF2*	ASD_29	meSNV; not maternal, parental sample n.a.	0.592/0.990	Reduced/OE	446–1241	0.0011–0	0.264; Inf (0.07-Inf)	C
cg25781162	chr2:44065997	*ABCG5*	ASD_9	meSNP MAF<0.004; P	0.429/0.869	Reduced	858–1713	0.0023–0	0.2529; Inf (0.08-Inf)	

*Region (DMRs)*	*Position (hg19)*	*Patient*	*REF_ID*	*Meth. avg. ASD/CNT*	*Pyroseq. meth. validation*			*RNAseq expression*	*Reported in ASD*
					*Validation/ segregation*	*case/parent AVG.*	*MAF ASD-CNT*		
*DMRs and replication of the epigenetic alterations*									
*AS1-SMG7; SMG7*	chr1:183439365–183439493	ASD_23	cg27513517, cg15632228	0.517/0.057	Hypermethylation; M inherited	43.98/45.05	0.008-0	*SMG7* (OE)	C
*PIK3CD; BC038541*	chr1:9747263–9747772	ASD_25	cg19309580, cg03653801	0.514/0.052	Hypermethylation; P inherited	39.18/49.81	0.008-0	*PIK3CD* (mild OE)	R, C
*PHACTR1*	chr6:13274151–13274354	ASD_25	cg06879394, cg21538684, cg20827128, cg07912922, cg00460589	0.415/0.049	Hypermethylation; P inherited	34.36/71.29	0.008–0.004	not altered	E, C
*RNU5E;RNU5D;ZCCHC9*	chr5:80597143–80597828	ASD_13	cg10569335, cg05602479, cg16993440, cg27010645, cg22211395, cg26543232, cg07673182, cg01036296, cg22439198, cg01491225, cg00407623, cg16288993	0.428/0.036	No sample available	—	—	*ZCCHC9* (DR)	C, L
*RAB26*	chr16:2198479–2198777	ASD_14	cg23462861,cg08813207	0.36/0.094	Hypermethylation; M inherited	—	—	*RAB26* NE,*PKD1* (mild DR)	C, L

Abbreviations: ASD, autism spectrum disorders; CNV, copy number variant; CI, confidence interval; DMR, differentially methylated regions; DR, downregulated; E, expression; L, linkage analysis; M, maternally inherited; MAF, minor allele frequency; mDS, micro deletion syndromes; Meth. avg. ASD/CNT, methylation average ratio of all CpGs in the region between the ASD case and the controls; n.a., not available; OE, overexpressed; OR, odds ratio; P, paternally inherited; Pyroseq.meth, Pysrosequencing method; R, related diseases; SNV, single-nucleotide variant.

a Statistically significant *q*-value (FDR correction).

b Statistically significant nominal *P*-value.

c Closest transcription starting site gene name.
