# Supplementary material for: Genetic and epigenetic methylation defects and implication of the ERMN gene in autism spectrum disorders
Source: Transl Psychiatry. 2016 Jul 12;6(7):e855–. doi: 10.1038/tp.2016.120 (PMC5545709; doi:10.1038/tp.2016.120)

Supplementary Figure 1. Manhattan clustering diagram for our cohort of ASD and control samples. ASD, autism spectrum disorder; C, controls.

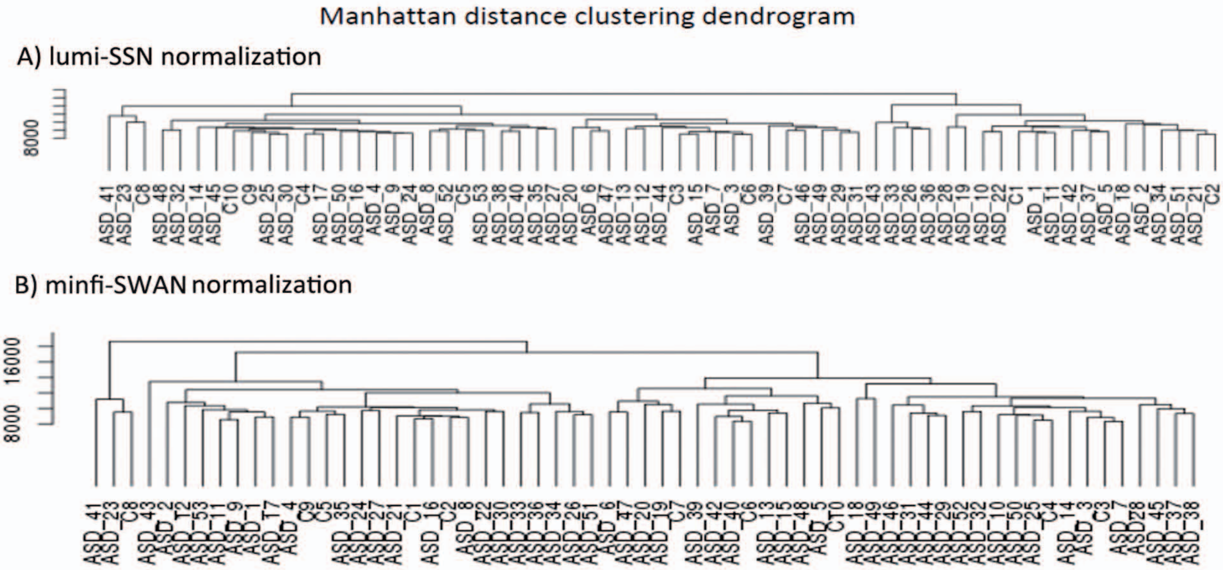

Supplementary Figure 2. Principal Component analysis for our cohort of ASD and control samples. ASD, autism spectrum disorder; CNT, controls.

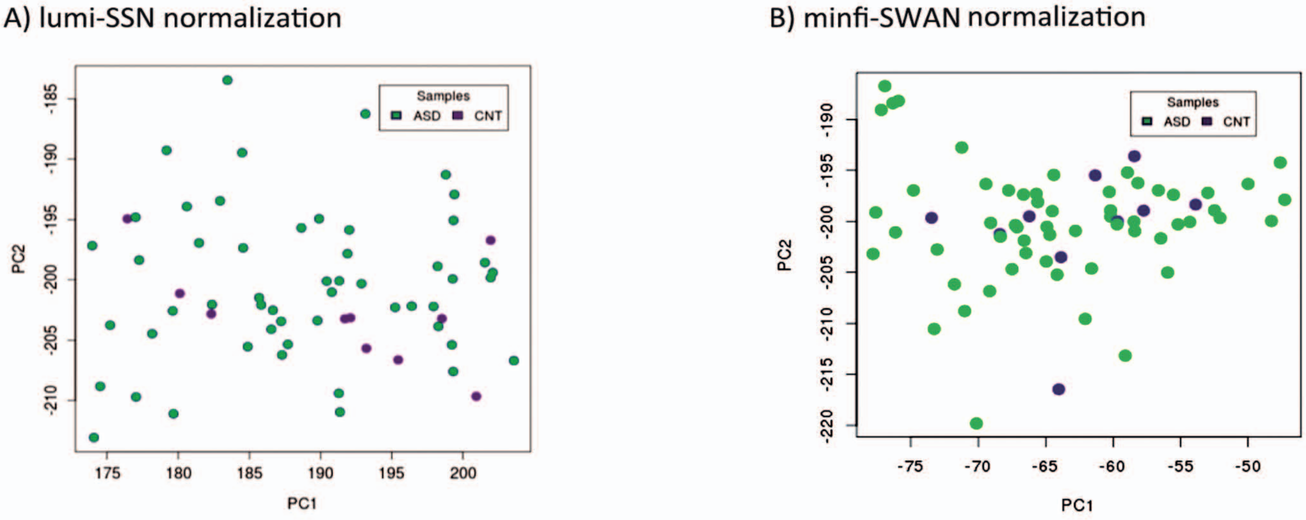

Supplementary Figure 3. Distribution by chromosome of 700DMCpG. ASD, autism spectrum disorder; CNT, controls.

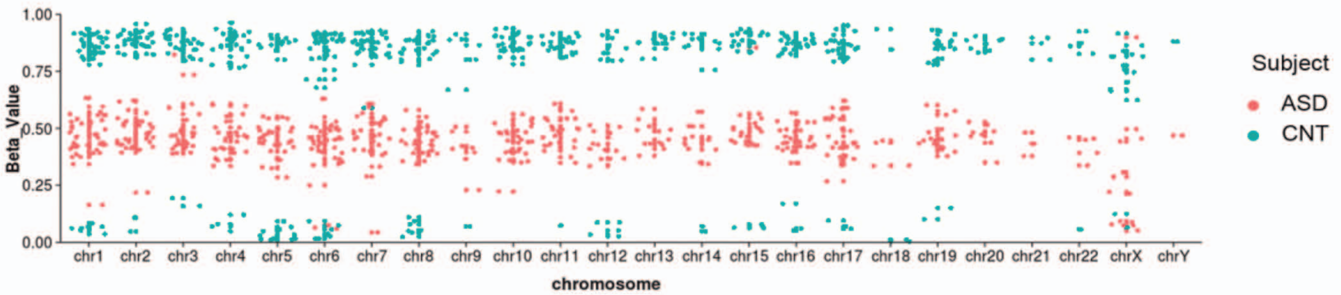

Supplementary Figure 4. Diagram showing the region, epigenetic variant validation, and expression for *PIK3CD* detected DMR. Relative hypermethylation in a clustered region in ASD_25 caused *PIK3CD* overexpression tendency. (**a**) Diagram for the region with the 2 CpGs altered pointed with an arrow. The diagram shows the genes in the region, the 450k array probes location, the DNAseI hypersensitive regions, and chromatin domains, being the alteration located in an active promoter region and an open chromatin region. (**b**) Beta values plot of the region divided by groups of the two CpG positions, showing for ASD_25 an increase methylation level and none of the controls with the alteration. (**c**) Pyrosequencing validation for the cg03653801 for the patient and parents. (**d**) Correlation of methylation values and expression values for ASD samples for *PIK3CD* gene for the CpGs studied. ASD, autism spectrum disorder; C, controls.

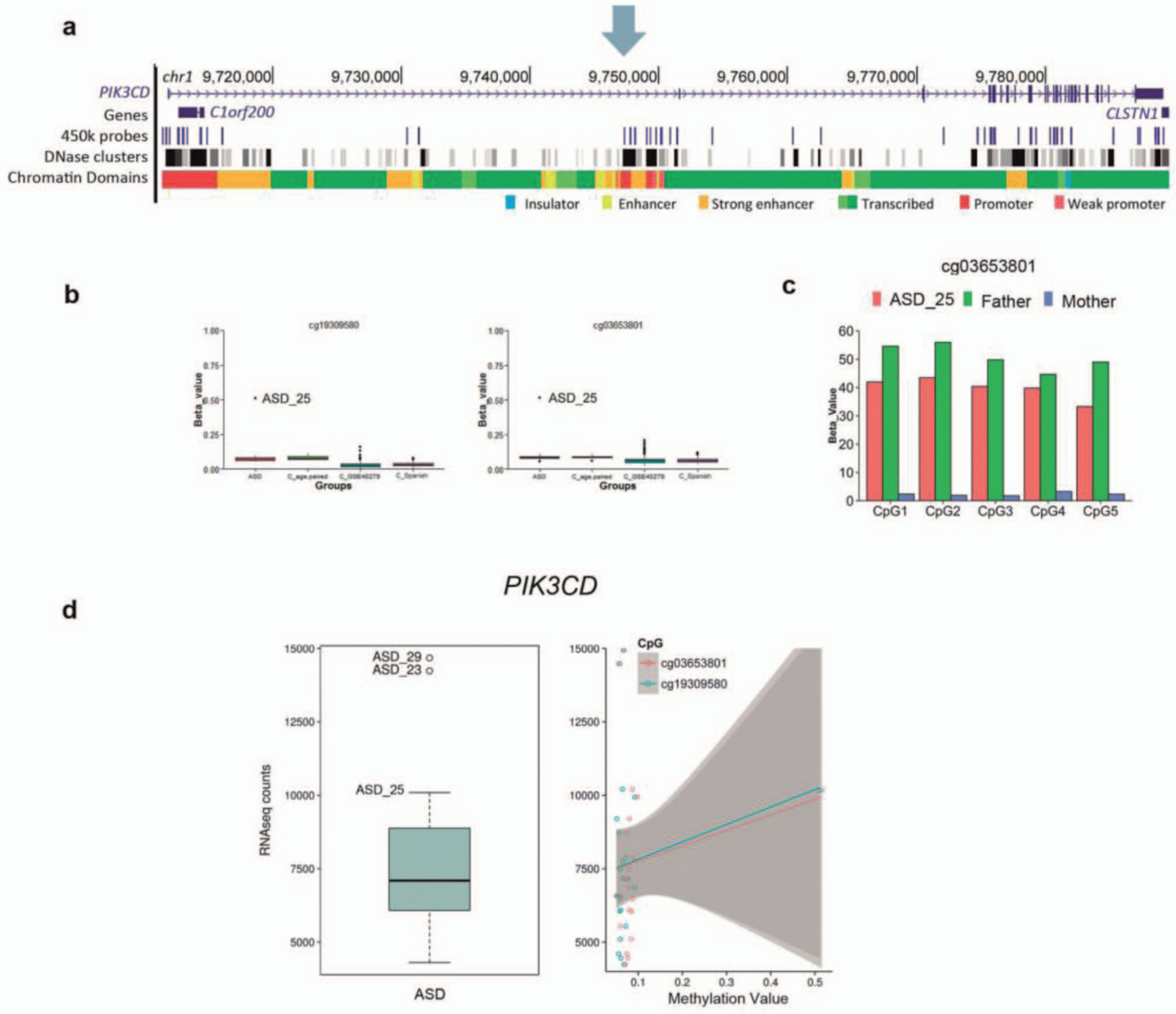

Supplementary Figure 5. Diagram showing the region and epigenetic variant validation for *PHACTR1* detected DMR. (**a**) The diagram shows the location of the 5 CpGs altered with an arrow, the genes in the region, the 450k array probes location, the DNAseI hypersensitive regions, and chromatin domains. Clustered CpGs were located in an active promoter in an open chromatin region. (**b**) Box plots for the beta values for the 5 CpGs by group (ASD, age paired controls and the Spanish and GEO (GSE:40279) controls set). (**c**) Pyrosequencing validation for the cg20827128. Expression levels for *PHACTR1* gene were not altered. ASD, autism spectrum disorder; C, controls.

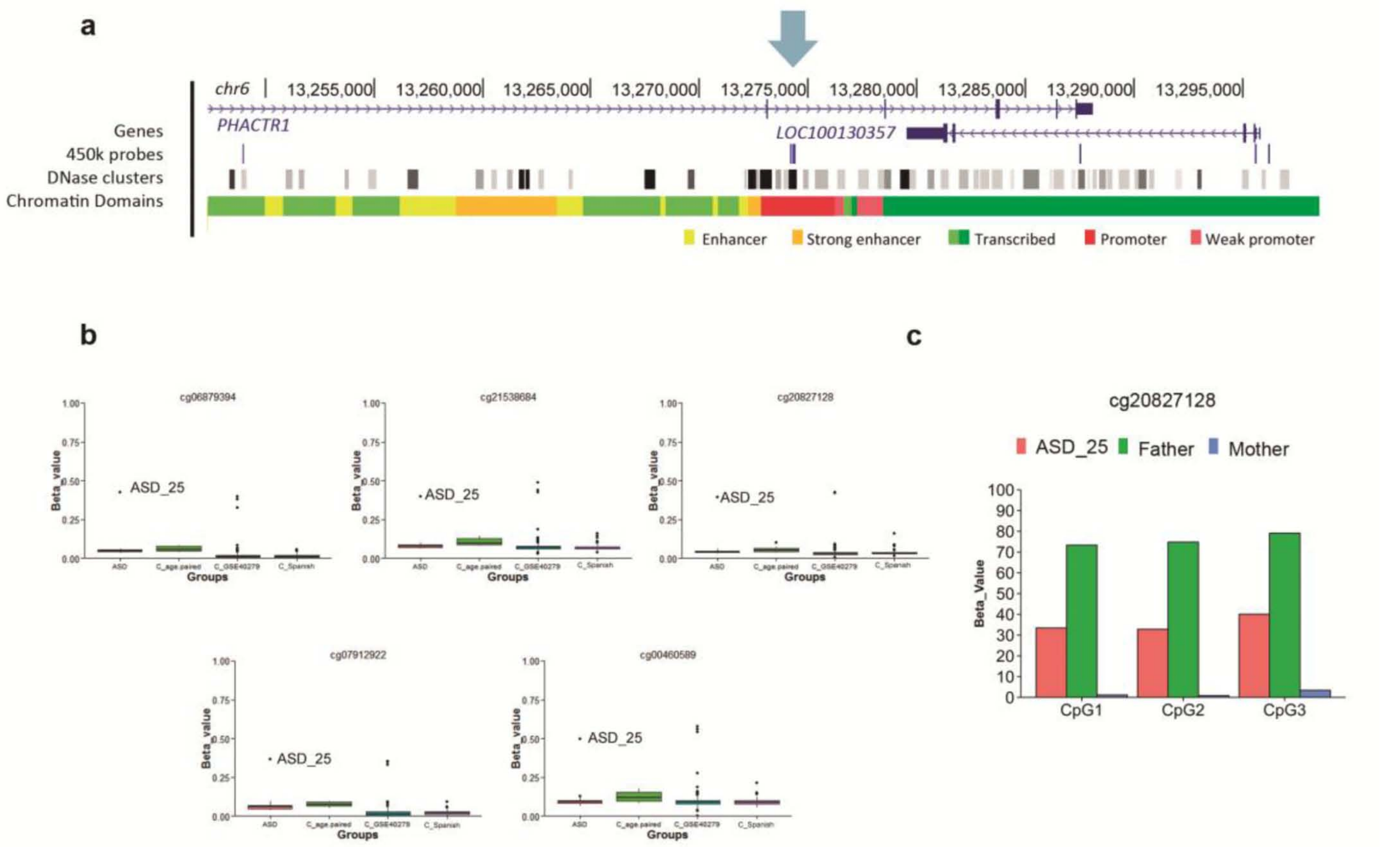

Supplementary Figure 6. Hypermethylation of clustered DMCpGs with an overexpression in *SMG7* in ASD_23. (**a**) Beta values across *SMG7* region showing the patient (green), the rest of the patients (red), the age-paired (blue) and the Spanish and GEO(GSE:40279) (purple) controls sets. Diagram under the graph shows the genes (SMG7 antisense and SMG7), 450k array probes, CpG Islands, DHSs and chromatin domains in the region. (**b**) Sanger Sequence of both CpGs showed no mutations. (**c**) Pyrosequencing validation revealed a maternal inherited hemimethylation for the patient. (**d**) A significant overexpression for *SMG7* gene and (**e**) correlation with methylation values was observed. ASD, autism spectrum disorder; C, controls.

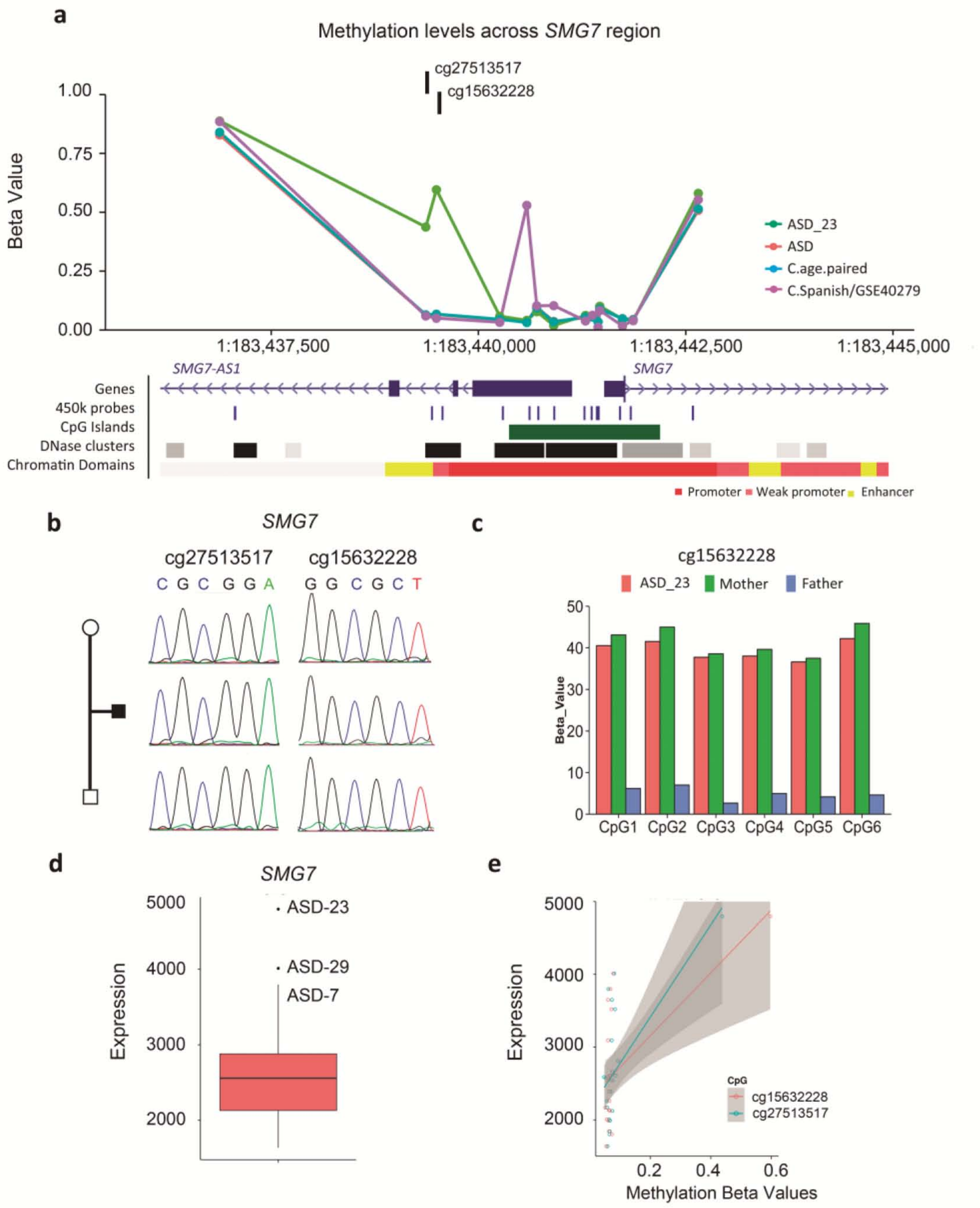

Supplementary Figure 7. Diagram showing the region, and expression for *RAB26* detected DMR. Clustered DMCpG region in *RAB26* showed an increase in methylation and a significant down-regulation in ASD_14. (**a**) Diagram showing the gene location, the 450k array probes, the DNAseI hypersensitive clusters, and chromatin domain regions. Two CpGs located in a CpG Island over *RAB26* promoter showed hypermethylation. (**b**) Expression for all ASD patients for the region. ASD_14 shows a down-regulation tendency for the *PKD1* gene. *RAB26* was not expressed in blood. ASD, autism spectrum disorder; C, controls.

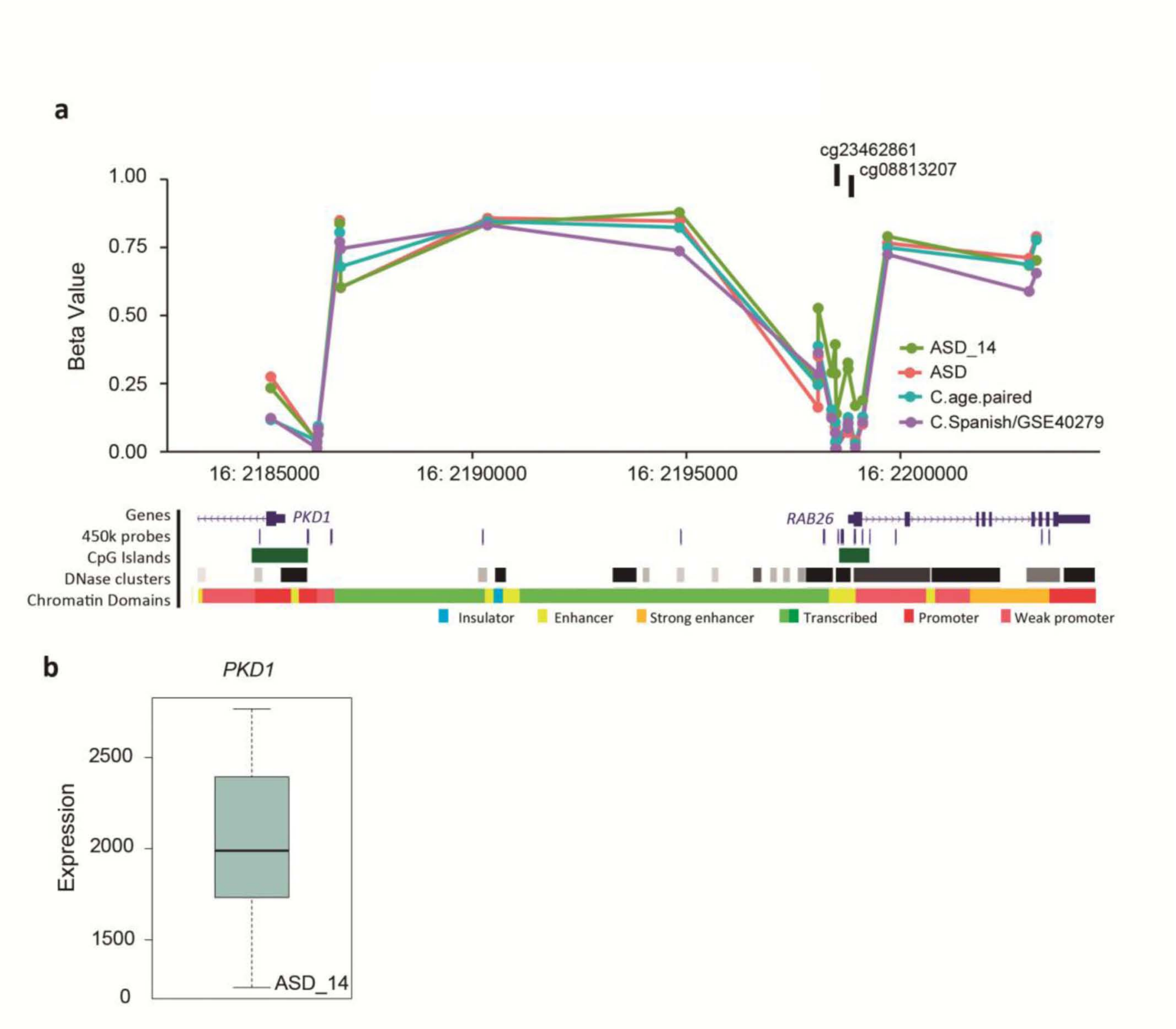

Supplement: Supplementary Figures 1–7 [file tp2016120x2.pdf]
