# Supplementary figures and images for: Genetic and epigenetic methylation defects and implication of the ERMN gene in autism spectrum disorders
Source: Transl Psychiatry. 2016 Jul 12;6(7):e855–. doi: 10.1038/tp.2016.120 (PMC5545709; doi:10.1038/tp.2016.120)

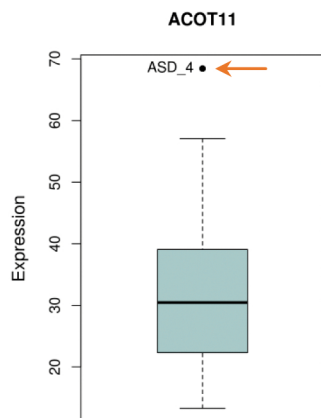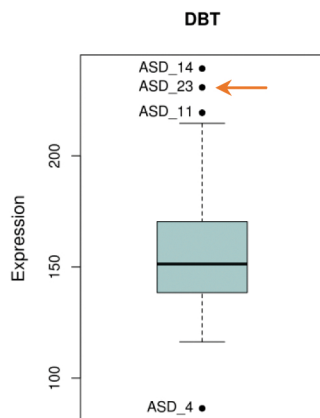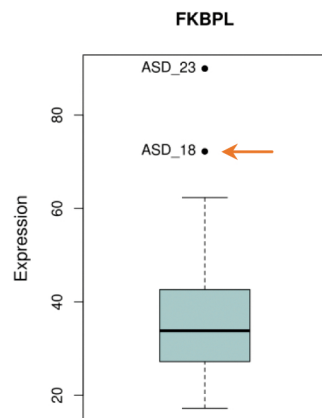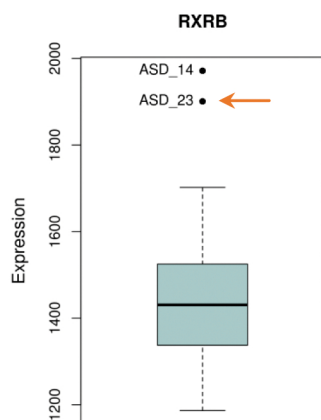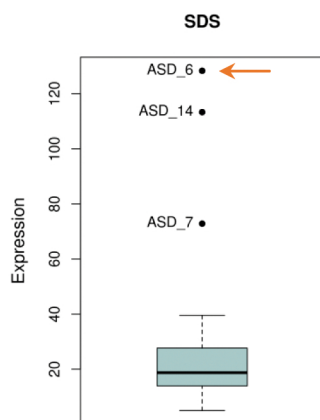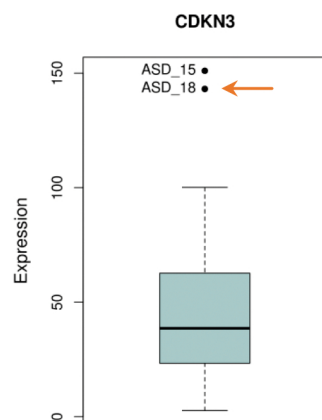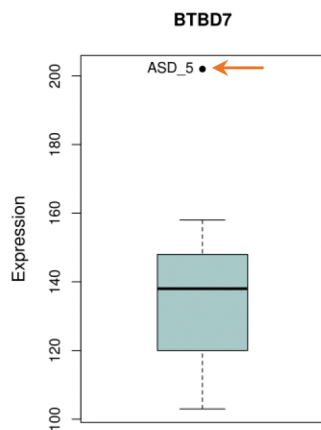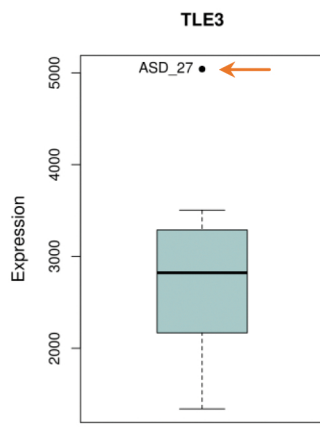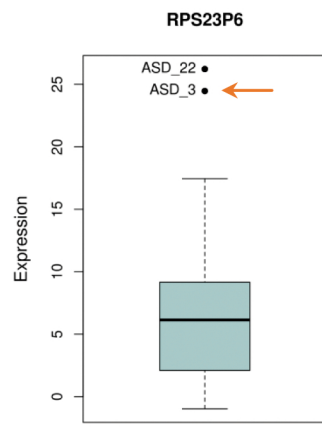

**SETD1A**

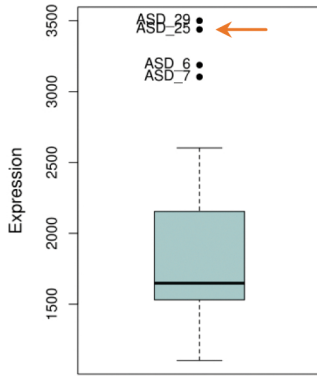

**STX1B**

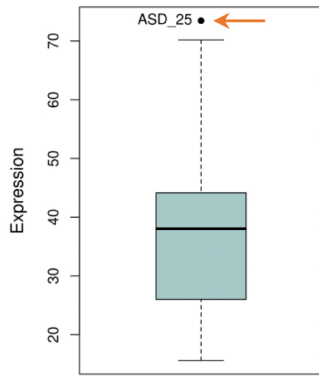

**CAMKK1**

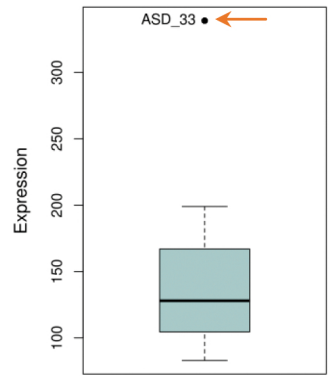

**MYO1F**

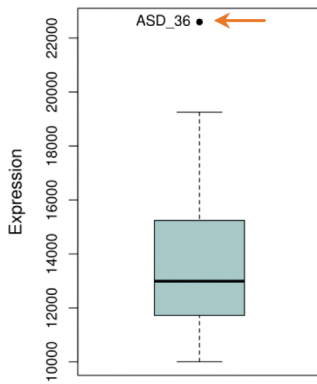

**ITPA**

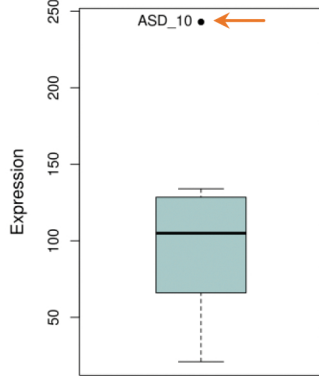

**SCARF2**

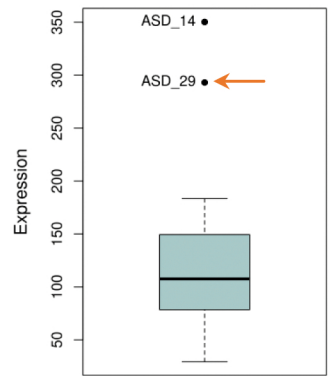

Supplement: Supplementary Figure 8 [file tp2016120x3.pdf]
